# From Gut Microbiota to Brain Waves: The Potential of the Microbiome and EEG as Biomarkers for Cognitive Impairment

**DOI:** 10.3390/ijms25126678

**Published:** 2024-06-18

**Authors:** Mahathi Krothapalli, Lauren Buddendorff, Hariom Yadav, Nathan D. Schilaty, Shalini Jain

**Affiliations:** 1 USF Center for Microbiome Research, Microbiomes Institute, University of South Florida, Tampa, FL 33612, USA; mahathik@usf.edu (M.K.); lbuddendorff@usf.edu (L.B.); hyadav@usf.edu (H.Y.); 2 Department of Neurosurgery and Brain Repair, University of South Florida, Tampa, FL 33612, USA; nschilaty@usf.edu; 3 Center for Neuromusculoskeletal Research, University of South Florida, Tampa, FL 33612, USA

**Keywords:** electroencephalography, microbiome, aging, Alzheimer’s, mild cognitive impairment, inflammation

## Abstract

Alzheimer’s disease (AD) is a prevalent neurodegenerative disorder and a leading cause of dementia. Aging is a significant risk factor for AD, emphasizing the importance of early detection since symptoms cannot be reversed once the advanced stage is reached. Currently, there is no established method for early AD diagnosis. However, emerging evidence suggests that the microbiome has an impact on cognitive function. The gut microbiome and the brain communicate bidirectionally through the gut–brain axis, with systemic inflammation identified as a key connection that may contribute to AD. Gut dysbiosis is more prevalent in individuals with AD compared to their cognitively healthy counterparts, leading to increased gut permeability and subsequent systemic inflammation, potentially causing neuroinflammation. Detecting brain activity traditionally involves invasive and expensive methods, but electroencephalography (EEG) poses as a non-invasive alternative. EEG measures brain activity and multiple studies indicate distinct patterns in individuals with AD. Furthermore, EEG patterns in individuals with mild cognitive impairment differ from those in the advanced stage of AD, suggesting its potential as a method for early indication of AD. This review aims to consolidate existing knowledge on the microbiome and EEG as potential biomarkers for early-stage AD, highlighting the current state of research and suggesting avenues for further investigation.

## 1. Introduction

Alzheimer’s disease (AD) is a neurodegenerative disease characterized by impaired cognitive function, and it is one of the most common causes of dementia [1]. Even in healthy aging, cognitive function does tend to worsen over time, but one’s ability to function independently should not be impaired, which is what occurs with AD [2,3]. Patients diagnosed with AD require assistance with daily activities such as eating, getting dressed, and managing medications [4]. During the progression of AD, the decline in cognitive function typically starts 20 years before diagnosis and occurs in stages. The initial symptomatic stage is mild cognitive impairment (MCI), which may progress to dementia [5,6]. Once the dementia stage is reached, preventative measures cannot be implemented, and symptoms cannot improve; thus, it is critical to detect AD in its early stages. The current methods of early AD diagnosis including cerebrospinal fluid analysis, blood tests, and PET scans are expensive and invasive. Therefore, developing a non-invasive method for an early AD diagnosis would be highly beneficial.

The microbiome is one potential marker currently under investigation. Recently, it has been found to be involved in several neurodegenerative diseases including AD, Parkinson’s disease, Huntington’s disease, amyotrophic lateral sclerosis (ALS), and multiple sclerosis [7,8,9,10,11,12,13,14,15,16,17,18,19]. The microbiota in the gut can influence brain health through the bidirectional relationship between the enteric nervous system and the central nervous system, known as the gut–brain axis [20]. There have been numerous studies supporting the presence of a relationship between the gut microbiome and AD [21,22,23]. Significant differences have been found between the microbiome signatures of individuals with AD and MCI and those of cognitively healthy individuals [21,22,23]. Furthermore, the transplantation of MCI/dementia host microbiomes accelerated cognitive decline within mouse models [24]. Similar to the gut microbiome, the oral microbiome signatures of those with AD and MCI also differed from healthy controls [25]. The mechanisms by which the microbiome contributes to MCI and ADRD (Alzheimer’s disease-related dementia) development still remain largely unknown; however, some studies show that an aged microbiota promotes gut permeability (“leaky gut”), which allows for the passage of inflammatory ingredients like lipopolysaccharide (LPS) and other antigens from the gut lumen into the circulation, increasing inflammation [26]. These abnormalities in the microbiome disrupt gut barriers (reduce mucin production), thus thinning the mucus layer, which in turn weakens tight junction proteins and exacerbates leaky gut [27,28]. Chronic leaky gut with low-grade inflammation increases the risk of cognitive decline and dementia. Additionally, dysbiosis of the gut microbiome can increase gut permeability, which subsequently can trigger systemic inflammation through the release of inflammatory cytokines [29,30]. Furthermore, the passage of the inflammatory cytokines through the blood–brain barrier (BBB) and into the brain results in neuroinflammation [29,30].

In recent years, electroencephalography (EEG) has emerged as a non-invasive tool to detect neuronal changes using electrodes placed on the scalp [31]. EEG has also been found to be associated with neurological disorders such as epilepsy, ADHD (attention-deficit hyperactivity disorder), and schizophrenia [5,32]. Recently, it has been utilized to help detect changes in brain activity related to different stages of Alzheimer’s disease (AD) or dementia [5,32]. While EEG patterns do change with normal cognitive aging, these patterns have been found to be distinct from those seen in MCI and AD patients [31,33,34,35,36,37]. Individuals with AD have been found to have an increase in delta and theta waves and a decrease in alpha and beta waves [31,38,39,40,41,42,43,44]. Individuals with AD have also been found to exhibit a decrease in EEG complexity and synchrony and overall EEG slowing compared to cognitively healthy individuals, indicating that EEG can be used as a biomarker [45,46,47,48]. In this article, we will discuss the potential of these new markers, EEG and the microbiome, in detecting AD risk during its early stages.

## 2. Cognitive Function in Aging and AD

Cognitive function tends to decline with age, posing a problem as the global aging population is increasing in both size and proportion [49,50,51]. The proportion of Americans 65 and older is projected to double to 22% by 2050 [52]. Aging is the greatest risk factor for AD, with 6.7 million Americans over the age of 65 suffering from AD-related dementia as of 2023 [53]. In normal cognitive aging, certain processes such as problem solving, reasoning, and memory skills steadily decline after the age of 65, but day-to-day life remains unhindered [2,3]. However, with AD-related dementia, there is a profound loss of cognitive function and those affected are unable to live independently. These symptoms not only affect those diagnosed with it, but they also place an economic and emotional burden on caregivers and family [54]. The annual cost of caring for someone with dementia in the United States is estimated to be USD 56,290, and due to a lack of methods for early detection, AD is not diagnosed until the dementia stage [55,56]. Early detection can help those affected prepare for the financial costs of Alzheimer’s disease (AD). Additionally, it provides the patient and their family with time to undergo counseling, process what will happen in the future, and devise a plan for how to cope with it. Moreover, early detection offers a better chance of slowing down the progression of the disease, as it enables the formulation of a treatment plan and facilitates lifestyle changes [57]. Finding a non-invasive way to detect AD earlier can alleviate some of the burdens that the caregivers and family of those affected face.

Studies suggest that structural changes in the brain occur during aging [2,58]. The brain decreases in size due to a loss of gray and white brain matter [2,58]. Most of the gray matter loss occurs in the prefrontal cortex, the region responsible for executive functioning [2,58,59]. White matter shrinkage is predominantly observed in the frontal lobe and around the corpus callosum, an area important for cognitive processes [2,58,60]. This loss of brain matter leads to synaptic loss—a major contributor to dementia [58,61]. Dementia occurs when synaptic loss compared to healthy adults is 40% [61]. ADRD specifically is caused by an increase in amyloid beta plaques and neurofibrillary tangles called tau in the brain, which leads to inflammation, neuronal death, and disrupted brain function [2,62,63]. Since AD is a progressive disease, neuronal damage occurs over time, and it can take years to develop dementia after the initial onset. The first stage, the preclinical stage, is asymptomatic but is indicated by amyloid beta deposition and an increase in tau [64]. The preclinical stage can last up to 30 years and not everyone advances to the next stage, which is mild cognitive impairment (MCI) [64]. MCI is characterized by cognitive issues that do not significantly interfere with day-to-day life [65]. The annual conversion rate of progression from MCI to dementia is currently between 10 and 15% [64,65,66,67]. For patients still in the MCI or early dementia stage, anti-amyloid medication can be used to slow down progression, but present symptoms cannot be reversed. Aducanumab and Lecanemab are treatments that have been approved by the FDA, but both require an official AD diagnosis before starting them. The current methods of AD diagnosis including cerebrospinal fluid analysis, blood tests, and PET scans can only detect the diseased state, and although accurate, they are expensive and invasive [68]. Additionally, many of these tests are associated with negative side effects such as radiation exposure from PET scans [68]. Finding a simple and non-invasive method that can be frequently administered for early AD or MCI diagnosis could provide an opportunity for those affected by AD to receive treatment that slows progression [68,69,70,71]. Furthermore, early detection biomarkers can be used to screen for individuals at a high risk of AD. Those individuals can then undergo further diagnostic testing, thereby increasing the probability of an early AD diagnosis.

## 3. The Role of the Microbiome in Aging and AD

In recent years, there has been a surge in research aimed at understanding the role of the microbiome in the gut–brain axis, including its potential as a biomarker for Alzheimer’s disease. The microbiome is the collection of bacteria, viruses, and fungi living in and on the body exerting a significant influence on human health. It inhabits different regions of the body, with a significant portion residing in the gut and oral cavities. Therefore, the gut and oral microbiomes have a considerable impact on human health [72]. These microbiomes directly impact health through the secretion of essential nutrients such as vitamins, essential amino acids, and lipids while also indirectly affecting the immune system and metabolic processes [73].

The diversity of the oral microbiome underscores its importance in cognitive health [74]. Chronic oral inflammation in conjunction with aging is identified as a risk factor for AD [75]. Many inflammatory oral diseases, including periodontitis, are influenced by the oral microbiome [75]. The oral microbiome is composed of 75 million bacteria belonging to 700 different species [76]. In addition to playing a role in inflammation, the oral microbiome also contributes to maintaining pH levels and inhibiting pathogen growth. Given the proximity of the oral cavity to the brain, the oral microbiome can also impact brain health by releasing inflammatory cytokines into the brain, contributing to neuroinflammation and AD development [75,77]. Several studies have highlighted variations in the composition of the oral microbiome between individuals with AD and cognitively healthy individuals correlating with AD severity [25,78,79,80]. For instance, as symptoms progress, there is a gradual increase in the abundance of Firmicutes and Fusobacteria phyla, accompanied by a decrease in the Proteobacteria phylum in AD individuals [25]. Moreover, AD patients demonstrate higher levels of pathogenic bacteria, including *Aggregatibacter actinomyctemcomitans*, *Porphyromonas gingivalis*, and *Fusobacterium nucleatum* [75,78]. *Aggregatibacter actinomyctemcomitans* is linked to the degradation of teeth-supporting tissues, while *Porphyromonas gingivalis* is a major contributor to chronic periodontitis and the destruction of periodontal tissue [75,81,82]. *Fusobacterium nucleatum* has been associated with both periodontal diseases and oral cancers [75]. Another study by Liu et al. (2019) found that AD patients showed an increase in the genera *Moraxella*, *Leptotrichia*, and *Sphaerochaeta* and a decrease in the genus *Rothia*, further suggesting the potential utility of the oral microbiome in AD detection [80]. Furthermore, differences were also observed between those with MCI and cognitively healthy individuals. Those with MCI had a lower abundance of the species *Gemella haemolysans* and *Streptococcus gordonii* and a higher abundance of *Veillonella* and *Fusobacterium* [79]. The observed variations in oral microbiome signatures between individuals with MCI and those with healthy cognition indicate a possible role for the oral microbiome in detecting early AD stages. Additionally, the link between AD and an increase in pathogenic bacteria underscores the importance of maintaining proper oral health to mitigate AD risk.

Gut microbiome alterations during aging significantly contribute to the development of ADRD. Research suggests that the composition and diversity of the gut microbiota can influence the aging process and contribute to the development of age-related diseases [83,84]. Microorganisms in the gut perform a variety of functions, including protection against pathogens, the activation of immune responses, vitamin production, and nutrients metabolism and extraction [72]. While the microbiome generally remains stable throughout life, it can be influenced by environmental factors such as stress, sleep, diet, smoking, exercise, and medication [85]. A healthy microbiome is characterized by high taxonomic diversity, but this diversity begins to decline around the age of 60 due to stress-induced imbalances in gut bacteria, a phenomenon known as gut dysbiosis [72]. More specifically, *Bifidobacteriaceae* and *Clostridium* abundance decreases with age, whereas the population of Proteobacteria increases [86]. This reduction in diversity, or gut dysbiosis, has detrimental effects on human health, characterized by altered host immune function, altered energy metabolism, and increased gut permeability, leading to increased intestinal and systemic inflammation. These conditions have been linked to various diseases such as diabetes, obesity, cardiovascular disease, cancer, and inflammatory bowel disease. More recent studies have also unveiled a connection between the gut microbiome and the development of several neurological disorders, including depression, multiple sclerosis, ALS, Parkinson’s disease, and AD [16,17,18,19,72,87,88,89]. The gut microbiome communicates with the brain through the gut–brain axis, a bidirectional communication pathway that integrates the gut’s functions with the cognitive and emotional centers of the brain [20,85]. This communication occurs through various mechanisms including the vagus nerve, the immune system, and bacterial metabolites [90]. The vagus nerve extending from the brain stem into the abdomen plays a significant role in signaling between the gut and the brain, modulating the central nervous system [85,91,92]. It receives stress signals from the gut, regulates the inflammatory cytokines produced by macrophages, and contributes to systemic inflammation [93]. Additionally, amyloid beta or tau present in the colon can travel to the brain via the vagus nerve, exacerbating AD [30]. There is also evidence that bacterial amyloid proteins present in the gut can incite an immune response, leading to the production of amyloid beta in the brain, thereby increasing the risk of developing AD [94].

Gut microbiome abnormalities contribute to the development of AD through gut dysbiosis, which leads to chronic low-grade inflammation [95]. Specifically, imbalances in the gut microbiota trigger the release of inflammatory cytokines, inducing systemic inflammation [29,30]. Leaky gut, characterized by increased gut permeability, exacerbates inflammation by allowing pro-inflammatory substances such as pathogens, antigens, and endotoxins to enter the bloodstream and lymphatic system, perpetuating chronic inflammation [96]. Aging, associated with decreased bacterial diversity, increases the likelihood of developing leaky gut [94,95,97,98]. Both gut dysbiosis and leaky gut can compromise blood–brain barrier permeability, possibly allowing microbes and cytokines from the gut to pass through to the brain and cause neuroinflammation [90,98,99,100]. Neuroinflammation exacerbates AD progression by increasing the formation of neurofibrillary tangles [101,102]. Probiotics, known for their beneficial effects on the gut, may mitigate AD symptoms during early stages [23]. For instance, individuals with MCI exhibited improved cognition after receiving the probiotic *Lactobacillus rhamnosus*, accompanied by a decrease in *Prevotella* bacteria abundance, indicating cognitive enhancement [23]. Recent research suggests that Bifidobacterium and lactic acid bacteria can reduce inflammation by secreting anti-inflammatory factors [103]. Enhancing microbiota diversity can mitigate leaky gut, thus reducing neuroinflammation. Studies demonstrate that microbiota diversity can increase within a day of dietary changes, highlighting diet’s pivotal role in mitigating gut dysbiosis and subsequently decreasing neuroinflammation [104]. For example, adherence to the Mediterranean diet positively influences the gut microbiome composition in individuals with MCI [97].

**Figure 1:**
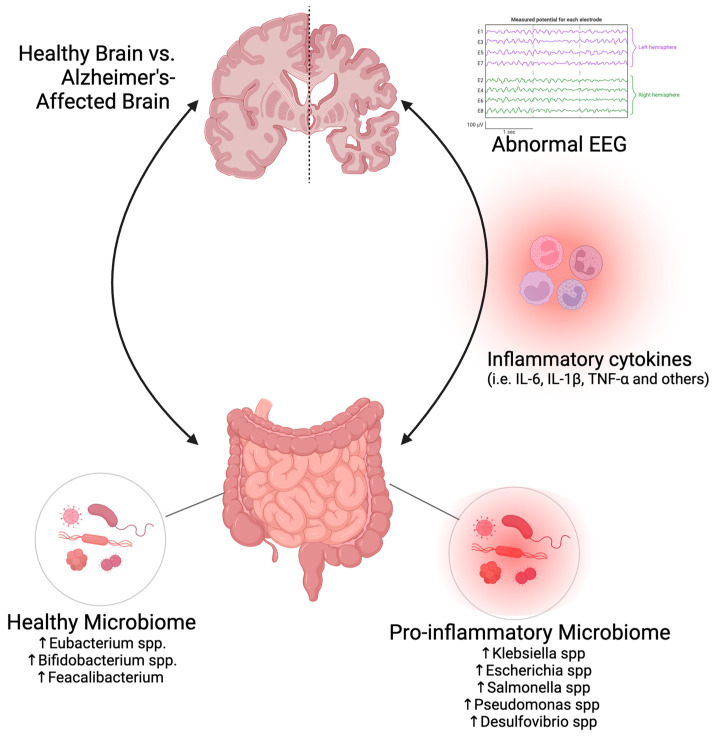
Potential AD biomarkers: EEG recordings in combination with a microbiome signature can be used as a potential biomarker for early detection of AD. Specific frequency characteristics and pattern changes observed in an EEG and microbiome (oral cavity and gut) signature in individuals with AD compared to cognitively healthy individuals. The brain and gut are connected through the gut–brain axis, and gut dysbiosis can induce inflammation in the gut, producing cytokines such as IL-6, IL-1β, TNF-α, and others released into the blood circulation, potentially causing neuroinflammation. An AD gut harbors a higher abundance of bacteria such as *Klebsiella*, *Escherichia*, *Streptococcus*, *Salmonella*, and *Pseudomonas* species, while a healthy gut harbors a higher abundance of anti-inflammatory bacteria, such as *Eubacterium*, *Bifidobacterium*, and *Feacalibacterium* spp.

The association between Alzheimer’s disease (AD) and the gut microbiome is apparent from the distinct microbiome signatures observed in those affected by AD. Vogt et al. (2017) discovered that individuals with AD exhibited a reduced microbial diversity and displayed an overall distinct and abnormal microbiome signature compared to the control group [21]. Specifically, participants with AD showed a decrease in the abundance of Firmicutes and Actinobacteria phyla along with an increase in the Bacteroidetes phylum [21,22]. This finding is supported by a recent study by Coradduzza et al. (2023), which revealed significantly different microbiome signatures in individuals in the early stages of AD compared to the control group [23]. Bacteroidetes and Firmicutes were the predominant phyla observed in individuals diagnosed with Alzheimer’s disease or dementia. The observed differences in signatures were associated with the presence of amyloid beta and tau in AD participants, suggesting that gut microbiome abnormalities influence brain health [23]. In addition, an AD/ADRD gut harbors a lower abundance of beneficial anti-inflammatory bacteria, such as *Eubacterium* spp., *Bifidobacterium,* and *Feacalibacterium* spp., compared to a healthy gut [21,56,105,106,107,108,109], while there is a higher abundance of several bacteria such as *Klebsiella*, *Escherichia*, *Streptococcus*, *Salmonella,* and *Pseudomonas* species that secrete functional amyloid proteins with a demonstrated capacity to cross-seed and trigger a cascade of amyloid protein misfolding [109,110] (Figure 1). Also, an AD gut is also enriched with Gram-negative bacteria that are a rich source of lipopolysaccharide (LPS), a cell wall component of Gram-negative bacteria, which can lead to increased production of pro-inflammatory cytokines, microglial priming, neuroinflammation, and neurodegeneration [111,112]. Additionally, pro-inflammatory taxa, such as the sulfate-producing *Desulfovibrio,* have been observed in AD patients [22]. Along with microbiome changes, gut microbial metabolites play an important role in AD and have been studied as potential biomarkers. The gut microbiome produces metabolites through bacterial metabolic processes, influenced by gut bacteria and diet [113,114]. These metabolites, including short-chain fatty acids (SCFAs), aromatic amino acids (AAAs), and Trimethylamine N-oxide (TMAO), can impact the nervous system by entering the circulatory system or crossing the blood–brain barrier (BBB) [113,114,115,116,117,118]. With age, the risk of metabolites affecting the nervous system increases due to a heightened gut dysbiosis risk and diminished BBB integrity [118].

SCFAs, the most prevalent gut metabolites, are primarily produced through dietary fiber fermentation [113,114,115]. SCFAs (butyrate and acetate) regulate pro-inflammatory cytokine activity by binding to immune cell receptors and G-protein-coupled receptors [113,115]. In addition to their beneficial effect on gut health, they have been found to play a role in improving brain function by modulating neuroinflammation, as they are able to cross the blood–brain barrier (BBB) [113,114,115]. In fact, they modulate BBB formation and synaptic plasticity [113,115]. SCFAs have also been implicated in Alzheimer’s disease (AD), as SCFA levels are negatively correlated with amyloid beta levels in AD patients [115], with SCFAs inhibiting amyloid beta aggregation and improving brain function. [115]. Moreover, dietary supplementation with SCFAs has been found to improve brain function, including improved memory and decreased neuroinflammation [114,115]. Aromatic amino acids (AAAs), specifically tryptophan, tyrosine, and phenylalanine, are produced as byproducts of microbial metabolism [113]. They serve as precursors for secondary metabolites and can be fermented to yield products such as ammonia, indole, and phenol [113,119]. Indoles, in particular, play a crucial role in regulating gut integrity and inhibiting neuroinflammation [114]. AAA metabolites are capable of crossing the blood–brain barrier (BBB), and studies have found that AD and MCI patients exhibit decreased indole levels and elevated amino acid levels [114,116]. Specifically, tryptophan and its secondary metabolites can inhibit enzymes involved in the formation of amyloid beta, a key factor in AD pathology [120]. Furthermore, kynurenine, a metabolite of tryptophan, can cross the BBB and form kynurenic acid and quinolinic acid, both of which are associated with cognitive decline [114,121].

Trimethylamine N-oxide (TMAO) is generated from choline and L-carnitine and is implicated in dementia [113,114,121]. Both choline and TMAO levels increase with age, which correlates with a higher risk of age-related diseases, including dementia [118]. TMAO is capable of crossing the blood–brain barrier (BBB), where it can cause synaptic damage, potentially leading to dementia [114,122]. It also induces the expression of the dementia marker CD68, contributing to neuronal aging [113,123]. Elevated TMAO levels are associated with reduced neurite density, a factor in cognitive impairment [117]. Additionally, TMAO has been found to cause the aggregation of amyloid beta and tau protein in the brain, which are key contributors to Alzheimer’s disease (AD) [114,124]. Studies indicate that patients with AD and mild cognitive impairment (MCI) exhibit elevated TMAO levels in their cerebrospinal fluid (CSF), urine, and blood compared to cognitively healthy individuals [113,114,117]. The microbiome’s metabolites represent one pathway through which it can impact the brain. These metabolites can be detected in the blood or cerebrospinal fluid (CSF), making them potential biomarkers for the microbiome. Thus, microbiome-derived metabolites can be considered promising candidates for promoting brain function and attenuating inflammation in AD.

Taken together, the alterations observed in both gut and oral microbiomes of individuals with AD, along with the link between gut inflammation and neuroinflammation, indicate that the microbiome holds potential as a biomarker for AD. The identification of microbiome signatures is relatively non-invasive, involving the collection of saliva and stool samples [125]. However, further research on the microbiome is imperative before solely relying on its use as a biomarker. One potential approach could involve combining the microbiome with another non-invasive biomarker for more comprehensive AD diagnosis and monitoring.

## 4. EEG as a Biomarker in AD

EEG, or electroencephalography, is a non-invasive and cost-effective method of measuring electrical activity in the brain by placing electrodes on the scalp. Brain waves are recorded in specific frequency bands during cognitive events such as sleep, balance, movement, and memory tasks [126]. (Table 1). Typically, EEG recordings involve 10 to 20 electrodes, but this number can be increased depending on the area of interest [127]. Power measures, which indicate the amount of activity in certain frequency bands, are commonly used to interpret EEG results [128].

EEG testing is versatile and easily repeatable, making it suitable for screening individuals at risk of developing Alzheimer’s disease (AD). Individuals suspected of having dementia during an EEG screening can then be referred for further testing. EEG has been widely employed in clinical settings to diagnose various neurological disorders, including epilepsy, sleep disorders, and encephalopathy [43,126]. In recent years, EEG has demonstrated its ability to differentiate between AD, MCI, and cognitively healthy individuals. Various EEG measures such as power distribution, spectral power ratio (SPR), complexity, and coherence have shown variations between AD patients, MCI subjects, and healthy controls [45,46,47,48]. An overall slowing of EEG activity in the frontal, temporal, parietal, and occipital regions of the brain has been observed in AD patients, characterized by a reduction in the power in high-frequency bands and an increased power in low-frequency bands [47,130,131,132,133]. Individuals with AD and MCI typically exhibit decreased activity in the alpha and beta frequency bands and increased activity in the delta and theta frequency bands [31,38,39,40,41,42,43,44,48,132,134,135,136,137]. This pattern contrasts with healthy aging, which is associated with a decrease in delta and theta waves [31,33,34,35,36,37,43,132,135]. Furthermore, reductions in alpha power have been correlated with the severity of dementia, with AD patients exhibiting a significant decrease compared to age-matched MCI control groups [41,42,132]. The spectral power ratio (SPR), which represents the ratio between fast and slow power frequencies, is also altered in individuals with MCI and AD [45,46]. AD is indicated by a low SPR compared to age-matched healthy controls [45]. A lower SPR has been associated with decreased cognitive function, enabling discrimination between MCI and AD groups, as MCI individuals exhibit a higher SPR than those with AD [45]. The beta/theta ratio has shown promising results in differentiating between AD, MCI, and healthy cognition with high sensitivity, allowing for the establishment of a cut-off for the MCI diagnosis [46]. EEG complexity and coherence, which reflect synchrony between cortical regions, have also been found to be reduced in AD patients [47,48]. These findings suggest that EEG holds potential for detecting AD in its early stages, as an increase in slow-frequency bands without a significant decrease in fast-frequency bands combined with a lower SPR can indicate MCI [41,45,46,134,136]. Gamma wave frequencies were also found to be altered in patients with AD [129,138,139]. A recent study by Traikapi and Konstantinou (2021) found disruptions in the gamma waves in AD patients and discovered that gamma stimulation could potentially reduce the severity of AD symptoms and slow cognitive decline [129]. Additionally, a study by Stothart et al. (2021) showed that EEG detected a significant difference in the recognition memory between AD patients and healthy older adults when they were tested using a fastball memory assessment [140].

EEG has also demonstrated both sensitivity and specificity in distinguishing between different types of dementia [57,141]. Frontotemporal dementia (FTD), which often presents with the same clinical symptoms as AD, frequently leads to misdiagnosis [141,142]. This is problematic as FTD and AD necessitate different prognoses and treatment plans [142]. However, studies utilizing EEG have successfully differentiated between AD and FTD subjects: individuals with FTD lack the EEG slowing characteristic of AD, while AD subjects exhibit less synchronization in fast frequencies (alpha and beta) [141]. Although both FTB and AD subjects show a decrease in alpha power, AD subjects demonstrate significantly higher theta power [143]. Dementia with Lewy bodies (DLB) is another common form of dementia often misdiagnosed due to its similarities with AD [57]. Early diagnosis of DLB is crucial, as antipsychotic medication is typically used to manage visual hallucinations—a symptom more common in DLB than AD [57]. Patients with DLB exhibit greater EEG slowing compared to those with AD [144]. Specifically, DLB subjects experience widespread EEG slowing throughout the brain, occurring earlier in disease progression than in AD [144]. Additionally, DLB subjects show reduced alpha power in the occipital lobe compared to AD patients. [57]. Moreover, DLB individuals exhibit decreased EEG connectivity and greater EEG abnormalities during the MCI phase, allowing EEG to differentiate between AD and DLB in the early stages of dementia [57]. Current diagnostic methods for distinguishing between dementia types such as PET scans and MRI are invasive, expensive, and time-consuming [57]. EEG offers a faster and less invasive alternative for the diagnosis of dementia.

Overall, EEG could play an important role in detecting and classifying dementia due to its ability to detect rhythm abnormalities in individuals with MCI and AD. Despite these promising findings, EEG is not routinely used for AD assessment in clinical settings due to limitations such as small sample sizes and limited population representation in existing data sets [5,43,135]. Therefore, EEG can complement other biomarkers, such as microbiome signatures, to enhance the accuracy of early AD diagnosis [5]. Additional research and validation are necessary to fully leverage the potential of EEG in AD diagnosis and classification.

## 5. Future Directions

The microbiome and EEG have not yet been used in clinical settings as biomarkers for Alzheimer’s disease (AD). Further studies with larger sample sizes and wider demographics are needed to validate both the microbiome and EEG as reliable AD biomarkers. We propose that EEG and the microbiome can be used in combination to provide a thorough and accurate AD diagnosis. Additionally, we suggest using the microbiome and EEG as screening tools. As both EEG and microbiome signatures are non-invasive and inexpensive diagnostic tools, they can be used to identify individuals at high risk of AD. These individuals can then undergo further testing for the diagnosis of AD, thereby increasing the chances of detecting AD in its early stages.

## 6. Conclusions

Alzheimer’s disease, a neurocognitive condition with severe consequences, underscores the need to impede its progression at its earliest stages. Present diagnostic methods for AD pose challenges in early detection due to their invasive and expensive nature. This review explores the potential of both the microbiome and EEG as biomarkers for the detection of AD. Recent years have witnessed an upsurge in microbiome research, revealing a link between the gut microbiome and the brain through the gut–brain axis. Distinct oral and gut microbiome patterns have been identified in cognitively healthy individuals and those with MCI or AD. Concurrently, EEG has gained recognition as a diagnostic and potential biomarker for assessing dementia severity, offering comprehensive insights into brain activity. Although research on utilizing EEG for dementia screening has been extensive, its integration into routine practice remains limited, and further studies with larger and more diverse sample sizes are needed. A major limitation regarding the use of EEG for AD diagnosis is the lack of studies including participants diagnosed with other dementia types. EEG changes can be observed in other types of dementia and neurological disorders, reducing the specificity of using EEG alone for a definitive AD diagnosis. Additionally, there is a lack of longitudinal studies that follow participants over time, testing the specificity and sensitivity of EEG in detecting the progression from the MCI stage to AD. Furthermore, the medications taken by AD patients should be considered, as antidementia drugs may impact study results. Another limitation is that EEG primarily captures electrical activity on the scalp, which may not fully represent deeper brain structures involved in AD pathology. Additionally, artifacts from muscle activity, eye movements, and environmental noise can interfere with the EEG signal, complicating data interpretation. However, the microbiome, in collaboration with EEG, emerges as a promising avenue for non-invasive biomarkers in the early stages of AD. Further exploration of their interconnection in detecting AD is essential to unravel their full potential as biomarkers.

## Figures and Tables

**Table 1:** The characteristics of different brain waves as well as the changes in brain activity associated with an AD diagnosis.

Brain Wave	Brain Activity	Frequency (Hz)	Change Observed in Aging	Change Observed in MCI	Change Observed in AD
Delta	Deep sleep	0.5 to 4	Decrease [31]	Increase [31]	Increase [39]
Theta	Initial stage of sleep, deeply relaxed	4 to 8	Decrease [31]	Increase [31]	Increase [39]
Alpha	Relaxed and attentive	8 to 13	Slight decrease [31]	Slight decrease [31]	Significant decrease [39]
Beta	Active, anxiety-dominant	13 to 30	No change [31]	No change [31]	Decrease [39]
Gamma	High cognitive function, concentration	30 to 80	No change [31]	No change [31]	Change observed [129]
